# How declines in fertility rates are informing responses within health systems: a protocol for a scoping review

**DOI:** 10.1136/bmjopen-2026-122442

**Published:** 2026-10-12

**Authors:** Tatjana Gibbons, Ektoras Georgiou, Becky Skidmore, Devon Evans, Stuart Gietel-Basten, Ester Lazzari, Lone Schmidt, Soren Ziebe, G David Adamson, Christian Malte Becker, Gitau Mburu

**Affiliations:** 1 University of Oxford Medical Sciences Division, Oxford, UK; 2 Department of Gynaecology, Fertility and Obstetrics, Rigshospitalet, Copenhagen, Denmark; 3 Department of Obstetrics, Gynaecology and Newborn Health, The University of Melbourne - Parkville Campus, Melbourne, Victoria, Australia; 4 Melbourne City IVF Fertility Clinic, Melbourne, Victoria, Australia; 5 The Royal Women’s Hospital, Parkville, Victoria, Australia; 6 Independent Information Specialist, Ottawa, Ontario, Canada; 7 Department of Obstetrics, Gynecology, and Reproductive Sciences, University of Manitoba, Winnipeg, Ontario, Canada; 8 Division of Social Science, The Hong Kong University of Science and Technology, Kowloon, Hong Kong, People's Republic of China; 9 Department of Demography, University of Vienna, Vienna, Vienna, Austria; 10 Department of Public Health, University of Copenhagen, Copenhagen, Denmark; 11 Fertility Physicians of Northern California, Palo Alto, California, USA; 12 Endometriosis CaRe Centre, Nuffield Department of Women’s and Reproductive Health, University of Oxford, Oxford, Oxon, UK; 13 World Health Organization, Geneva, Switzerland

**Keywords:** Subfertility, Reproductive medicine, GYNAECOLOGY, Health policy

## Abstract

**Abstract:**

**Introduction:**

Global total fertility rates (TFR) are declining by approximately 1% annually due to factors such as increasing use of contraceptives, urbanisation, a shift in social norms, educational and socioeconomic changes among others. The result is a growing proportion of the world population not meeting their family-building intentions, a shift in global demographic profiles, an ageing population with different social and health system needs and a reduced workforce and taxpayer base to support economic demands. How health systems across the globe are responding to this ‘fertility transition’ has not yet been systematically mapped.

**Methods and analysis:**

The proposed scoping review will follow the Arksey and O’Malley framework and will be reported in accordance with the Preferred Reporting Items for Systematic reviews and Meta-Analyses extension for Scoping Reviews (PRISMA-ScR). Data will be obtained through a comprehensive search of academic databases and grey literature. All documents describing health systems’ responses to fertility decline will be considered. Responses will be coded using Braun and Clarke’s inductive and deductive analysis approach and the types of responses will be mapped within the WHO Building Blocks of Health Systems. Analysis will determine how health system responses vary across regions, cultures and TFR levels, and how they intersect with policies outside the health system, including pronatalist family policies.

**Ethics and dissemination:**

This review is being commissioned by the WHO’s Department of Sexual Reproductive, Maternal, Child, Adolescent and Ageing Health as part of WHO’s efforts to enable the promotion and protection of people’s health as it relates to sexuality and reproduction, as well as the implementation of sound public health strategies. Results will be disseminated through submission to a peer-reviewed scientific article, the WHO website and will be used by the WHO to inform and promote further public health adaptations to declines in TFR. Ethical approval is not required.

**STRENGTHS AND LIMITATIONS OF THIS STUDY**

- The scoping review is based on a comprehensive peer-reviewed database search strategy and a grey literature search.
- The grey literature search will examine each country using translated terms to develop a comprehensive understanding of health system responses and adaptations to the decline in total fertility rates (TFR) worldwide.
- Despite the grey literature search strategy being language-tailored, there is a risk of missing eligible documents due to limitations in ChatGPT’s translation and Google search algorithms can restrict results by location.
- There are inherent challenges in searching for policy documents, as many documents, discussions and actions are not publicly available, potentially limiting the inclusion of certain documents.
- Global responses and adaptations to the decline in TFR will be mapped within the WHO Health System Building Blocks, enabling a systematic comparison.

## Introduction

 A majority of nations across the globe are experiencing a decline in their population’s fertility rates.^1^ This global health phenomenon has attracted the attention of policymakers due to its significant socioeconomic and geopolitical implications. The total fertility rate (TFR) is a metric that describes the average number of children a woman would have between the ages of 15 and 49 if current age-standardised fertility rates were maintained throughout her reproductive lifespan.^2^ The United Nations (UN) estimates show that global TFR declined from 5.0 in 1960 to 2.2 in 2024. Additionally, global TFR is projected to reach replacement level (2.1 per woman) by 2050, and to continue to fall thereafter.^3^

The cause of this decline is multifactorial, encompassing individual, socioeconomic and health-related factors. The primary driver of this decline is women’s education and increasing use of contraceptives. In addition, urbanisation, technological advances and shifts in social and family ideals have contributed to higher education rates and increased participation of women in the workforce, which ultimately leads to an increased mean age at childbearing. Moreover, a cultural shift is believed to be occurring, leading to greater acceptance and desire for smaller families, including couples choosing not to have children. The acceptance of and desire for a smaller family are influenced by many factors, including ethical concerns about raising children in the current climate and growing economic pressures (often referred to as the ‘cost of living crisis’).^4 5^ However, many who reach the end of their reproductive years are left wishing they had larger families, often due to barriers, such as socioeconomic factors and infertility, that prevent individuals from realising their fertility goals.^6 7^

Infertility affects approximately 17.5% of the adult population,^8^ and although infertility is not the most significant driver of declines in TFR,^5^ it is impacted by the shifts in social behaviours such as delayed childbearing, as advancing maternal and paternal age is linked to declining gamete quality. Ageing oocytes accumulate DNA damage, accompanied by a reduced capacity to detect and repair damage, resulting in increased aneuploidy rates.^9^ Over time, sperm quantity and quality are believed to decline.^10^ Environmental pollutants have also been investigated as a potential cause of infertility. Although evidence is inconclusive, microplastics, environmental xenoestrogens and other endocrine-disrupting chemicals are believed to affect fecundity in both males and females.^11–13^ Currently, fertility procedures such as in vitro fertilisation and intracytoplasmic sperm injection account for around 2–5% of births in some countries.^14 15^ However, there are global discrepancies in the availability of such services.^16 17^

Contemporary declines in TFR as part of ‘fertility transitions’^16^ and a shift in the demographic profile are a global concern, as they are accompanied by an ageing population with different health systems and social needs, and a reduced workforce to support these needs. As such, various family policies, including pronatalist policies, have been introduced by governments in response to declining TFRs, such as family benefits, childcare support, parental leave and other labour market reforms.^5^ These responses are crucial to target the socio-economic factors and drivers that exist beyond health systems. However, alone, they are unable to reverse the TFR declines.^5^ Other systems, such as healthcare, also play a crucial role in responding to the declines in their populations’ TFR by providing crucial adaptations needed to support the changing population structure. As such, there is growing consensus that responses from various sectors must not exist in isolation, given the wide variety of contributing factors.^15^ Therefore, understanding how responses intersect is vital to how countries adapt to the demographic changes we face.

The WHO Health System Building Blocks^18^ identify the six essential components of a health system: service delivery, health workforce, health information systems, access to essential medicines, health financing and leadership and governance. These building blocks enable the mapping of how health systems are responding to socio-economic shifts through the development and implementation of actions, policies and initiatives. The WHO Health System Building Blocks have previously been used in reviews to describe factors affecting the implementation of fertility services within health policy in regions such as The Gambia^19^ and China.^20^

A systematic assessment of how countries are responding to declines in TFR within the health sector has not yet been mapped. Therefore, the proposed scoping review will systematically identify and map countries’ responses to declines in TFR within the WHO building block domains. Moreover, it will assess how these responses are informed by TFR trends by mapping the types of data used to develop them, the stakeholders driving these changes, how the effectiveness of these responses is measured and what they tell us about the priorities of countries experiencing a TFR decline. It will also assess whether these responses and priorities differ regionally, culturally, economically or by the decline in TFR. Finally, the review will assess how existing types of health system responses intersect with other social policies implemented in response to the decline in TFR.

Although research into this topic is limited, the proposed review will build on similar projects, for instance, the 2024 International Federation of Fertility Societies (IFFS) consensus document^21^ on the implications of the declining global fertility rates on family planning. The IFFS consensus document highlighted the importance of reproductive policies in addressing declining TFR and the shift from traditional policies aimed at controlling population growth toward policies that promote ‘family building’. It also highlighted policies implemented to improve fertility awareness and increase access to fertility care services. To broaden the findings of work such as this, the proposed scoping review will assess responses across all WHO building blocks of health systems, and extend beyond academic bibliographic indices (eg, PubMed), to include comprehensive grey literature searches, allowing for the inclusion of other relevant high-level government policy documents, such as acts, laws, interparliamentary discussions, policy progress reports and commissioned reports that are not readily available in academic databases.

In summary, the scoping review aims to provide insights into how health systems are responding to declines in TFR, how these responses are informed, and how they differ across regions, offering actionable guidance for health stakeholders globally to adapt their reproductive health policies within the continually evolving landscape.

## Methods and materials

The review will be conducted using Arksey and O’Malley’s^22^ scoping review framework and later iterations of the framework by the Joanna Briggs Institute and Levak *et al*.^23^ This approach outlines a transparent, rigorous and iterative process for searching, charting, mapping and analysing data. Data will be reported according to the Preferred Reporting Items for Systematic Reviews and Meta-Analyses extension for Scoping Review checklist (PRISMA-ScR),^24^ including the use of a PRISMA flow diagram of included studies (figure 1). Please see online supplemental file S2 for the PRISMA-ScR.

**Figure 1:**
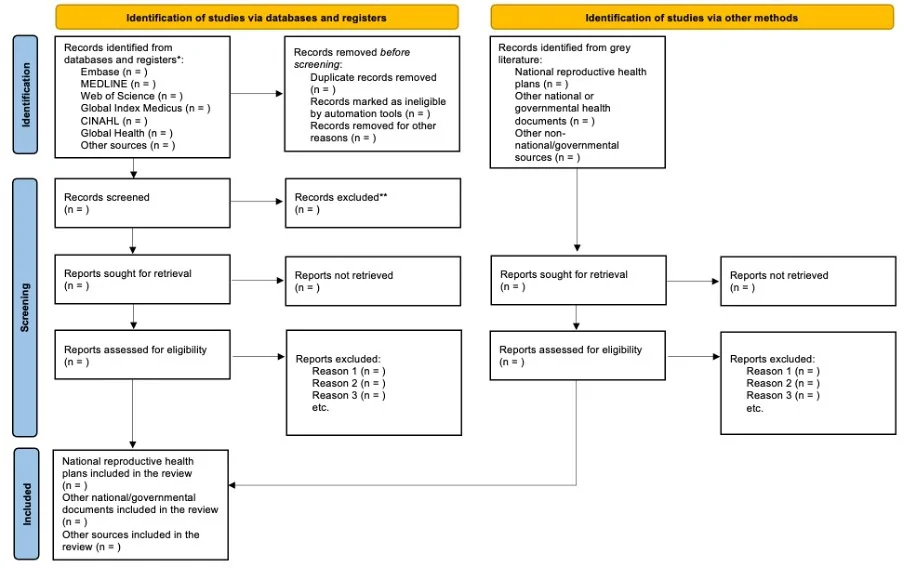
PRISMA flow chart of included studies. Expected flow chart to be used to describe the inclusion and exclusion process of the identified records. PRISMA, Preferred Reporting Items for Systematic Reviews and Meta-Analyses.

A scoping review design was chosen, allowing for a broad mapping of available actions and initiatives from a variety of academic and non-academic sources, described without the restrictive framework of a systematic review. Within this design, the thematic analysis would enable the expression of a narrative about the current responses and priorities of health systems. The review will be based on the methodology of two successful scoping reviews that mapped the global availability of endometriosis policies and guidelines.^25 26^ The project is commissioned by the WHO’s Human Reproduction Programme and aligns with its objectives of mapping and promoting health system policies responsive to global demographic shifts, including the fertility transition. By systematically mapping national responses, the review aims to uncover disparities in adaptations to TFR declines and identify areas for targeted future initiatives, thereby supporting progress toward the WHO’s objective of Universal Health Coverage.

In March 2026, preliminary searches were conducted in Google Scholar, the Open Science Framework Registry, the Cochrane Database of Systematic Reviews, JBI Evidence Synthesis and PROSPERO to ensure that no published or ongoing scoping reviews addressed the same research questions. In PROSPERO’s registry, a protocol for a systematic review of strategies for implementing health policies to promote fertility was registered.^27^ The protocol was deemed to be suitably different in terms of its methodology (systematic review, as opposed to a scoping review), focus (fertility promotion policies, rather than a focus on health system responses to declining TFRs), outcomes (implementation outcomes eg, acceptability and feasibility, rather than a focus on the types of responses within WHO building blocks stratified by region, culture and TFR) and search strategy (grey literature search limited to WHO, United Nations Population Fund, and national ministries of health websites, with the addition of country (and language) specific searches of responses in Google and Google Scholar included within this protocol).

### Identifying the research questions

#### Primary research question

The JBI Population, Concept, Context (PCC) framework was used to develop the primary review question.

1. In populations facing a fertility decline (population), how are health system responses informed by these declines (concept) across WHO-recognised regions (context)?
  - How can these responses be mapped to the WHO health system building blocks?
  - What are the sources of information used to inform health system responses?

#### Secondary research questions

1. Who are the key stakeholders or agencies driving these responses?
2. How is the effectiveness of these responses measured?
3. What are the identified barriers to implementing and actioning health system responses that are informed by declining fertility rates?
4. How do responses and priorities of health systems vary across geographical, cultural or economic profiles, and how are these differences related to the levels of TFR?
5. How do the types of responses that exist intersect with broader family policies (eg, pronatalist policies) that exist in response to declining fertility rates?

### Identifying relevant literature

#### Eligibility criteria

The eligibility criteria are based on the PCC framework (table 1).

**Table 1:** Inclusion criteria of the scoping review using the PCC framework

PCC	Inclusion criteria	Exclusion
Population	All national or subnational populations experiencing a fertility rate decline. Nations not experiencing a fertility decline will not be described within the WHO Health system building block analysis but will be included within how responses relate to levels of TFR.	Data will not be excluded based on country.
Concept	All types of health system responses and broader family policies (eg, pronatalist policies) that have been informed by declining TFRs. Responses include actions, policies, initiatives, strategies, reforms and adaptations. Health system responses will be described within the WHO building blocks of health systems. Therefore, health system responses include, but are not limited to: Governance and leadership. Health financing. Service delivery. Access to essential medicines and technologies. Health workforce and training initiatives. Health information, education, awareness and surveillance. Included responses include those which are stated to be a consequence of declining TFRs, for instance health system adaptations for an ageing population. Responses not pertaining to health systems will not be described within the WHO health system building blocks analysis but will be included in the analysis of how health system responses intersect with other family policies (eg, pronatalist policies).	Responses not described in relation to declining TFRs or resulting demographic changes will be excluded.
Context	International, national, territorial or subnational settings (eg, states, provinces or territories) or regions stratified by TFR.	Responses without identifiable international, national, territorial or subnational relevance will be excluded. Responses by individuals or local hospitals are likely to be excluded but noteworthy local policies (as agreed by the authorship group) may be presented separately.
Types of evidence sources	Any source referring to health system responses, including but not limited to peer-reviewed primary or secondary research (eg, reports, or academic publications), policy documents, government, UN agencies (eg, WHO) or NGO (eg, academic societies), such as reports. Multiple sources referring to the same response can be included so long as they address different aspects of the research questions.	Personal opinion pieces, informal commentary and sources without identifiable governmental or national/international NGO affiliation will be excluded.

NGO, non-governmental organisation; TFR, total fertility rate.

#### Database search

The database search was developed by a senior information specialist (BS) in consultation with the lead author (TG). The complete search strategy is available in (online supplemental file 1). The MEDLINE search will be peer-reviewed by another experienced information specialist using the Peer Review of Electronic Search Strategies (PRESS) tool. Comments will be addressed prior to finalising and translating the strategy to the other databases. Using the multifile option and deduplication tool on the Ovid platform, we will search Ovid MEDLINE ALL, Embase, Global Health and Social Policy and Practice. We will also search CINAHL on EBSCO, Web of Science (Core Collection), and Global Index Medicus (all indexes). Strategies will use a combination of controlled vocabulary (eg, “Fertility”, “Birth Rates”, “Family Planning Policy”) and keywords (eg, “total fertility rate”, “fertility decline”, “falling birth rate”, “policy”), with no language restrictions on any of the searches. However, the searches will be limited to publication years from 1990 to the present, and, where possible, animal-only records will be removed. The search was restricted to literature published from 1990 onwards, as, given the duration of the reproductive lifespan, policies introduced since 1990 are more likely to reflect countries’ responses to current fertility trends, reflecting reproductive behaviours of population cohorts that have not yet completed their lifetime fertility.

Files will be downloaded and deduplicated using EndNote V.9.3.3 (Clarivate) and then uploaded to Covidence software (Veritas Health Innovation), where TG and DE will independently screen them guided by the screening tool (figure 2). An initial test subset of titles and abstracts will be screened to mitigate any disparities in protocol interpretation. The two authors will resolve conflicts in the screening process; unresolved conflicts will be referred to GM.

**Figure 2:**
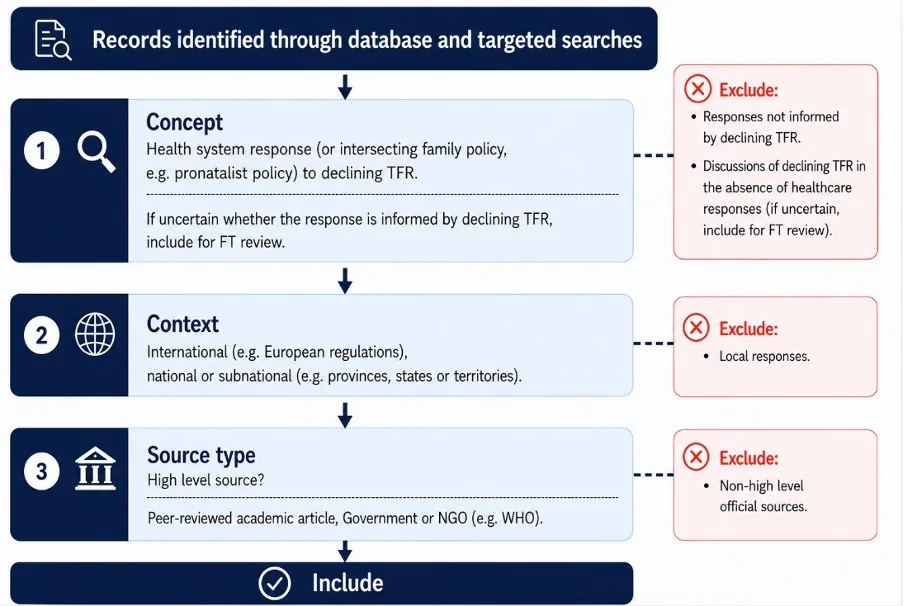
Database screening tool. The screening tool was developed to assist and standardise the database screening process for the two independent screeners FT, full text; NGO, non-governmental organisation; TFR, total fertility rates.

### Grey literature search

#### Step 1: searching for national reproductive health plans

The grey literature search will be country-tailored. The term ‘country’ is defined by the WHO’s list of countries and territories (all of which are members of the United Nations) as reported on the 5 May 2026 (n=194).^28^

A search will be conducted to identify the national policies of countries that describe their reproductive plans. The search included terms in the local language, translated by ChatGPT (model GPT-5.5). These documents will then be reviewed to locate responses to the declines in TFR. ChatGPT (model GPT-5.5) will serve as the default translation tool, except for websites or online references, where a built-in Google Translate plugin will be used to assist with screening for document relevance. In the event of ambiguous translations, the alternative translation tool will be used (eg, GPT first, followed by Google Translate) and then human translation will be sought.

Each country’s Department of Health (DoH) website will be searched with the following terms:

- ‘Reproductive health Plan’
- ‘National Health Plan’
- ‘Population Policy’

Google will be searched is national policies (Reproductive Health Plan, National Health Plan and Population Policy) are not identified on the country’s DoH website. The following terms will be used:

- “Country” + (“national health plan” OR “national health strategy” OR “reproductive health plan” OR “reproductive health strategy” OR “population policy”)
- The search will be conducted in English and the native language, and the first 50 results will be screened.

Google Scholar will be searched (if national policies are not identified on DoH or Google), using the following terms:

- national|fertility|family|reproductive|population+strategy|strategies|strategic|plan|plans+country
- The search will be conducted in English and the native language, and the first 50 results will be screened.

#### Step 2: searching for other high-level policy documents

The second phase will involve identifying health system responses in prominent policy documents, such as reports and laws, through comprehensive searches on Google and Google Scholar, as well as in selected resources. In some instances, the results of the database search may aid the grey literature search by highlighting policy initiatives. Google and Google Scholar searches will be conducted in English and the native language, using ChatGPT (GPT-5.5). The first 100 results will be screened (or until 20 consecutive irrelevant results are found).

Google Scholar search terms:

- fertility|“birth rate”|“birth rates”**+**replacement|decline|declining|decrease|decreasing|drop|dropping|fall|falling|sub-replacement|subreplacement**+**policy|policies|mandate|mandates|governance|response|responses+country

Google search terms:

- “Country” + “responses to total fertility rate decline”

Selected grey literature:

The selected grey literature will be screened for supplementary information on trends and responses to the decline in TRF. This part of the search will not be country-tailored. The following selected sources will be searched using terms such as “responses to total fertility rate decline”:

- United Nations Department of Economic and Social Affairs Population Division^29^
- United Nations Population Fund^30^
- Netherlands Interdisciplinary Demographic Institute^31^
- Institute for Health Metrics and Evaluation^32^
- Wittgenstein Centre^33^

### Data extraction and charting

Data from the included sources will be extracted into the data extraction table templates (both tables will be side by side in a single Excel sheet). All included documents will have table 2 completed, and sources pertaining to specific health system responses will also have table 3 data extracted. It is possible that an included source might be included as it supports a response, but does not specifically describe a response; thus, it will have data only extracted into table 2. The data extraction tables will allow for research questions 1a, 1b, 2, 3 and 4 to be addressed.

**Table 2:** Excel template for document referencing

Referencing								Summary
Country or region	Doc. ID	Title	Source type	Method of identification	Date published	URL	Last accessed or searched	Document description

**Table 3:** Excel template for health system response mapping

Health system response coding					
Response type ‘code’	WHO Health system building block	Stakeholders involved	Informed by	Effectiveness measure	Barriers identified

Data will be independently extracted by TG and reviewed by an additional author. Any disagreements that arise between the reviewers will be resolved through discussion or with GM. Authors or organisations of sources will be contacted to request missing or additional data where required, within reason. The draft data extraction tool will be iterated as necessary during data extraction from each included study. Iterations will be detailed in the full scoping review report.

### Data analysis

An inductive and deductive Braun and Clarke’s Thematic Analysis of the results will be conducted. The inductive analysis will involve generating ‘response type codes’ to describe the health system responses being developed, informed by the declines in fertility rates. For instance, in 2020, the Japanese government addressed declining TFR by expanding financial support for medically assisted reproduction (MAR)-related medications and insemination, shifting from a subsidy-based approach to full coverage under national health insurance. This response may initially be coded as ‘expansion of MAR subsidisation to full coverage’.^34^ Codes will be iterated and refined based on the data obtained. Similar codes will be clustered and collapsed to improve comparability. For instance, if many responses include full coverage for MAR but do not mention earlier subsidisation, the codes may be collapsed into ‘full coverage for MAR’. Codes will be given regardless of enforcement status, in recognition that implementing responses within health systems is a multistep process. For example, a bill and a law will be contained within the same code, as their primary intentions remain consistent. The codes will be analysed to detect recurring themes.

Following the primarily inductive thematic analysis, a deductive framework will be used to map and aggregate themes into the WHO Health System’s six core building blocks.^18^ This aggregation follows other authors’ work on describing fertility policies in health system,^19^ and prior work conducted by the first author on endometriosis policies.^25^

In the context of this scoping review, the building blocks can be described as follows:

1. Governance and leadership: This building block will discuss how health system responses are organised, planned, implemented and how the strategic planning and oversight is held accountable through monitoring practices. For instance, this building block will discuss data on the availability of responses to fertility decline in National Action Plans, also referred to as National Health Strategies or Policies.
2. Service delivery: This building block will include responses to develop a care system that offers timely access to high-quality services, including the expansion of specialised fertility services, pre-natal and pre-conception care and antenatal care. Other health system adaptations in response to the declining TFR and its implications will also be considered, for instance, responses that account for the increased need for support for the ageing population.
3. Health workforce: This building block will include initiatives that ensure that when patients seek support, they are met with a well-trained, informed and adequately distributed workforce.
4. Health information systems: Health systems rely on accurate information given and received. This building block will describe how health system responses are informed by health-related strategic information, data, research, monitoring and evaluation and auditing. It will also describe responses to promote a well-informed population and patient group (eg, through education).
5. Access to essential medicines: This building block includes responses to develop a care system that provides timely access to high-quality treatments.
6. Financing: Responses within this building block will include the financing of responses to address the decline in TFR and those that support universal access to care. As such, responses within the discussed code ‘full coverage for Assisted Reproductive Technologies (ART)’ would be included within this theme. To develop an understanding of financing trends coverage, codes for MAR and ART will not be collapsed.

The manuscript will be structured to describe the results identified within each of the WHO Health System Building Blocks. To address research questions 5 and 6, the results will be analysed to assess how they vary across various demographics:

- Geographical region (defined by WHO: African region, Region of the Americas, South-East Asia Region, European Region, Eastern Mediterranean Region and Western Pacific Region).^35^
- Economic classification (defined by the ‘World Bank Group income classifications for FY26’: not classified, low-, lower-middle-, upper-middle-, high-income).^36^
- Cultural or socio-political groupings (where relevant).
- TFR (lowest-low fertility ≤1.3, low fertility 1.3–2.0 and at or above replacement level ≥2.1).^37^

Finally, the themes and types of responses identified will be considered within the broader approach to addressing declining TFR, including how health system responses intersect with broader pronatalist policies identified in the database and grey literature search, but those not included within the analysis of health system responses. For instance, systematic reviews, narrative reviews, or non-government organisations/non-state actors reports on regional family policies (including pronatalist policies).^38–40^

## Ethics and dissemination

The scoping review protocol has been designed to guide the development of a comprehensive scoping review assessing health system responses and adaptations to the decline in TFRs. This is a novel topic that, to our knowledge, has not yet been comprehensively analysed within a scoping review framework. The results of this scoping review will be disseminated through a peer-reviewed scientific article and used by the WHO to inform and support stakeholders’ efforts in the development and promotion of sound public health policies. Given the iterative process of the scoping review design, revisions to the proposed methodology may occur, guided by the results obtained. Methodologically, the review may be impacted by reliance on one data extractor, due to feasibility and resource availability, but validation of key selected studies will be conducted. In addition, any modifications to the protocol will be described in the published review manuscript to enhance transparency. The scoping review does not require ethical approval or patient consent, as it will draw on published existing literature. Patient and public involvement was not considered necessary. The protocol for this scoping review has been registered on the Open Science Framework.

## Supplementary material

10.1136/bmjopen-2026-122442 online supplemental file 1

10.1136/bmjopen-2026-122442 online supplemental file 2
