# Supplementary material for: How declines in fertility rates are informing responses within health systems: a protocol for a scoping review
Source: BMJ Open. 2026 Oct 12;16(10):e122442. doi: 10.1136/bmjopen-2026-122442 (PMC13662019; doi:10.1136/bmjopen-2026-122442)
Supplement: Supplementary file 1 [file bmjopen-2026-122442.reviewer_comments.pdf]

PEER REVIEW HISTORY

BMJ Open publishes all reviews undertaken for accepted manuscripts. Reviewers are asked to complete a checklist review form and are provided with free text boxes to elaborate on their assessment. These free text comments are reproduced below.

ARTICLE DETAILS

Title (Provisional)

How declines in fertility rates are informing responses within health systems: A protocol for a scoping review

Authors

Gibbons, Tatjana; Georgiou, Ektoras; Skidmore, Becky; Evans, Devon; Gietel-Basten, Stuart; Lazzari, Ester; Schmidt, Lone; Ziebe, Soren; Adamson, G David; Becker, Christian Malte; Mburu, Gitau

---

VERSION 1 - REVIEW

**Reviewer**	**1**
**Name**	**Casilla-Lennon, Marianne**
**Affiliation**	**Yale University, Urology**
**Date**	**18-Jun-2026**
**COI**	**None**

This protocol discusses the important area of health policy related to the dramatic global fertility decline. The WHO building-blocks framework offers a coherent structure for evaluating this gap in the literature. Overall, the methodology is rigorous and follows established scoping-review conventions, with a peer-reviewed search strategy and pre-registration. My comments are confined to two areas of the methods where additional clarification would strength reproducibility. I recommend accept with minor revisions.

Minor revisions:

Grey literature search

- Although including grey literature is worthwhile to capture the policy and governmental documents central to the review’s aims. Two clarifications would strengthen the study design.

- The protocol describes machine translation, using Google Translate and ChatGPT, as interchangeable translation tools, whereas in practice they differ substantially in outputs and, more importantly, in reproducibility. It would help to state which tool serves as the

default and under what circumstances the other is used. When using an LLM, the model and version should be stated.

- In addition, the protocol for dealing with uncertain or ambiguous translations should be explicitly addressed (ex. Is a flagged document escalated to a human translator, re-checked or just excluded?)

Data extraction and charting

- The methodology would benefit from dual independent extraction (p. 12 line 54-56 suggest that data will be extracted by one researcher and then reviewed by a second) as opposed to an extract-then-verify method.

References

- Please review the references imbedded in the text and ensure they correspond correctly to those in the bibliography

---

VERSION 1 - AUTHOR RESPONSE

Dr. Marianne Casilla-Lennon's comments

This protocol discusses the important area of health policy related to the dramatic global fertility decline. The WHO building-blocks framework offers a coherent structure for evaluating this gap in the literature. Overall, the methodology is rigorous and follows established scoping-review conventions, with a peer-reviewed search strategy and pre-registration. My comments are confined to two areas of the methods where additional clarification would strength reproducibility. I recommend accept with minor revisions. Thank you very much for your comments and feedback.

Minor revisions:

1. Grey literature search - Although including grey literature is worthwhile to capture the policy and governmental documents central to the review's aims. Two clarifications would strengthen the study design.The protocol describes machine translation, using Google Translate and ChatGPT, as interchangeable translation tools, whereas in practice they differ substantially in outputs and, more importantly, in reproducibility. It would help to state which tool serves as the default and under what circumstances the other is used. When using an LLM, the model and version should be stated.
    - Added on page 10. The search included terms in the local language, translated by ChatGPT (model GPT-5.5). These documents will then be reviewed to locate responses to the declines in TFR. ChatGPT (model

GPT-5.5) will serve as the default translation tool, except for websites or online references, where a built-in Google Translate plugin will be used to assist with screening for document relevance. In the event of ambiguous translations, the alternative translation tool will be used (e.g. GPT first, followed by Google Translate), and then human translation will be sought.

2. In addition, the protocol for dealing with uncertain or ambiguous translations should be explicitly addressed (ex. Is a flagged document escalated to a human translator, re-checked or just excluded?)
    - As above
3. Data extraction and charting
   - The methodology would benefit from dual independent extraction (p. 12 line 54-56 suggest that data will be extracted by one researcher and then reviewed by a second) as opposed to an extract-then-verify method.
    - Unfortunately, given the team's constraints and the large number of sources to screen, this is not possible. We have added this as a potential limitation on page 15: Methodologically, the review may be impacted by reliance on one data extractor, due to feasibility and resource availability, but validation of key selected studies will be conducted.
4. References - Please review the references embedded in the text and ensure they correspond correctly to those in the bibliography.
    - Completed

VERSION 2 - REVIEW

**Reviewer**	**1**
**Name**	**Casilla-Lennon, Marianne**
**Affiliation**	**Yale University, Urology**
**Date**	**12-Aug-2026**
**COI**	

Revisions were made appropriately. Agree to accept.
