# Supplementary material for: How declines in fertility rates are informing responses within health systems: a protocol for a scoping review
Source: BMJ Open. 2026 Oct 12;16(10):e122442. doi: 10.1136/bmjopen-2026-122442 (PMC13662019; doi:10.1136/bmjopen-2026-122442)
Supplement: Supplementary file 2 [file bmjopen-16-10-s001.docx]

| 1 | exp Fertility/ and (replacement or declin* or decreas* or drop or dropped or dropping or drops or fall or falling or fallen or fell or reduc* or slump* or sub-replacement).ti. |
| --- | --- |
| 2 | ((fertile or fertility) adj3 (replacement or declin* or decreas* or drop or dropped or dropping or drops or fall or falling or fallen or fell or reduc* or slump*)).tw,kw,kf. |
| 3 | Birth Rates/ and (replacement or declin* or decreas* or deterior* or diminish* or dip or dipped or dipping or dips or drop or dropped or dropping or drops or dwindl* or fall or falling or fallen or fell or low or lower or lowering or reduc* or sag or sagged or sagging or sags or slump* or sub-replacement or wane or waning).ti. |
| 4 | (rate? adj2 (birth? or reproducti*) adj3 (replacement or declin* or decreas* or deterior* or diminish* or dip or dipped or dipping or dips or drop or dropped or dropping or drops or dwindl* or fall or falling or fallen or fell or low or lower or lowering or reduc* or sag or sagged or sagging or sags or slump* or sub-replacement or wane or waning)).tw,kw,kf. |
| 5 | ((demographic or population* or fertility rate? or total fertility rate? or TFR) adj3 (replacement or declin* or decreas* or deterior* or diminish* or dip or dipped or dipping or dips or drop or dropped or dropping or drops or dwindl* or fall or falling or fallen or fell or sag or sagged or sagging or sags or slump* or wane or waning)).tw,kw,kf. |
| 6 | (("no" or nil or null or zero) adj3 (population adj2 growth)).tw,kw,kf. |
| 7 | (childfree or child-free or childless or antinatalism or anti-natalism).tw,kw,kf. |
| 8 | or/1-7 [**DECLINING FERTILITY/INFERTILITY**] |
| 9 | Policy/ |
| 10 | Family Planning Policy/ |
| 11 | Health Policy/ |
| 12 | Public Policy/ |
| 13 | Social Control Policies/ |
| 14 | Politics/ |
| 15 | (policy or policies or politic?).ti,kw,kf. |
| 16 | (policy or policies or politic?).ab. /freq=2 |
| 17 | (mandate or mandates).tw,kw,kf. |
| 18 | ((family building or family planning or fertile or fertility or federal or government or health or healthcare or infertile or infertilit* or jurisdiction* or national or population or pronatal* or pro-natal* or provincial* or public or reproducti* or social control or state) adj3 (policy or policies or politic?)).tw,kw,kf. |
| 19 | (family policy or family policies).tw,kw,kf. |
| 20 | Government/ |
| 21 | Government Programs/ |
| 22 | Federal Government/ |
| 23 | State Government/ |
| 24 | Delivery of Health Care/lj, og [Legislation & Jurisprudence, Organization & Administration] |
| 25 | Delivery of Health Care/ and (adapt* or chang* or innovat* or respond* or respons*).ti. |
| 26 | Health Care Reform/ |
| 27 | ((country or "country's" or countries or family planning or fertile or fertility or federal or government or health care or healthcare or health system? or infertile or infertilit* or jurisdiction* or national* or policy or policies or politic? or population or pronatal* or pro-natal* or provincial* or public or reproducti* or social control or state or system-level) adj3 ((action adj2 plan?) or adapt* or blueprint? or chang* or initiative? or innovat* or priorit* or program or programs or programme or programmes or reform* or resilien* or respond* or respons* or roadmap? or road map? or strategy or strategies or strategic)).tw,kw,kf. |
| 28 | governance?.ti,ab,kw. |
| 29 | governance?.ab. /freq=2 |
| 30 | ((family building or family planning or fertilit* or pronatal* or pro-natal* or reproducti*) adj3 governance?).tw,kw,kf. |
| 31 | Jurisprudence/ |
| 32 | Legislation as Topic/ |
| 33 | (jurispruden* or law or laws or legal* or legislat* or statute or statutes).ti,kw,kf. |
| 34 | (jurispruden* or law or laws or legal* or legislat* or statute or statutes).ab. /freq=2 |
| 35 | Civil Rights/ |
| 36 | Human Rights/ |
| 37 | Reproductive Rights/ |
| 38 | ((fertile or fertility or infertile or infertility or reproducti*) adj2 right?).tw,kw,kf. |
| 39 | Right to Health/ |
| 40 | Women's Rights/ |
| 41 | right?.ti,kw,kf. |
| 42 | right?.ab. /freq=2 |
| 43 | or/9-42 [**POLICIES, PROGRAMMES, RIGHTS, ETC**.] |
| 44 | 8 and 43 [**DECLINING FERTILITY - POLICIES, PROGRAMMES, ETC**.] |
| 45 | "Global Burden of Disease"/ |
| 46 | "Cost of Illness"/ |
| 47 | Financial Stress/ |
| 48 | ((econom* or financ*) adj3 (challeng* or distress* or hardship* or pressur* or strain*)).tw,kw,kf. |
| 49 | or/45-48 [**FINANCIAL BURDEN/COST OF ILLNESS**] |
| 50 | 8 and 49 [**FERTILITY/INFERTILITY - FINANCIAL BURDEN/COST OF ILLNESS**] |
| 51 | Financing, Government/ |
| 52 | (Fertility/ or exp Infertility/) and (budget* or financ* or fund or funds or funding or incenti* or monetary support* or monies or subsid* or tax or taxes or taxation).ti,kw,kf. |
| 53 | ((fertility or infertile or infertility or subfertility or sub-fertility or subfecundity or sub-fecundity or reproducti*) adj5 (budget* or financ* or fund or funds or funding or incenti* or monetary support* or monies or subsid* or tax or taxes or taxation)).tw,kw,kf. |
| 54 | ((tax or taxes or taxation) adj2 (assistance? or benefit? or credit? or deduct* or incenti* or relief? or subsid*)).tw,kw,kf. |
| 55 | ((childbearing or child-bearing or fertile or fertility or infertile or infertility or reproducti*) adj2 (assistance? or benefit? or credit? or deduct* or incenti* or relief? or subsid*)).tw,kw,kf. |
| 56 | (public* adj (fund* or subsid*)).tw,kw,kf. |
| 57 | Insurance/ |
| 58 | exp Insurance Coverage/ |
| 59 | exp Insurance, Health/ |
| 60 | (insure? or insuring or insurance*).ti,kw,kf. |
| 61 | (insure? or insuring or insurance*).ab. /freq=2 |
| 62 | (insur* adj2 cover*).tw,kw,kf. |
| 63 | Health Benefit Plans, Employee/ |
| 64 | (employ* benefit? or health benefit? or benefit plan? or benefit scheme?).tw,kw,kf. |
| 65 | Managed Care Programs/ |
| 66 | (managed adj (care or health care or healthcare)).tw,kw,kf. |
| 67 | exp Social Security/ |
| 68 | Maternal Welfare/ |
| 69 | Social Welfare/ |
| 70 | ((maternal or social) adj3 (benefit? or security or subsid* or welfare)).tw,kw,kf. |
| 71 | or/51-70 [**FUNDING, SOCIAL BENEFITS/WELFARE/INSURANCE**] |
| 72 | 8 and 71 [**FERTILITY/INFERTILITY - SOCIAL BENEFITS/WELFARE/INSURANCE**] |
| 73 | Education/ |
| 74 | Education, Graduate/ or exp Education, Medical/ or exp Education, Nursing/ or Education, Continuing/ or Education, Public Health Professional/ or Internship, Nonmedical/ or Interprofessional Education/ or Mentoring/ |
| 75 | Sexology/ or Sex Counseling/ or Sex Education/ |
| 76 | Health Workforce/ |
| 77 | (educat* or mentor* or teach* or train*).ti,kw,kf. |
| 78 | (educat* or mentor* or teach* or train*).ab. /freq=2 |
| 79 | ((fertility or infertile or infertility or subfertility or sub-fertility or subfecundity or sub-fecundity or reproducti*) adj3 (educat* or mentor* or teach* or train*)).tw,kw,kf. |
| 80 | ((educat* or train*) adj2 ((action adj2 plan?) or adapt* or blueprint? or chang* or initiative? or innovat* or priorit* or program or programs or programme or programmes or reform* or respond* or respons* or roadmap? or road map? or strategy or strategies or strategic)).tw,kw,kf. |
| 81 | Health Education/ |
| 82 | Public Health/ed [education] |
| 83 | ((health or public) adj2 (aware* or educat* or information* or surveil*)).tw,kw,kf. |
| 84 | ((fertility or infertile or infertility or subfertility or sub-fertility or subfecundity or sub-fecundity or reproducti*) adj2 (aware* or educat* or information* or surveil* or track*)).tw,kw,kf. |
| 85 | or/73-84 [**AWARENESS, EDUCATION, TRAINING**] |
| 86 | 8 and 85 [**FERTILITY/INFERTILITY - AWARENESS, EDUCATION, TRAINING**] |
| 87 | Delivery of Health Care/ |
| 88 | Delivery of Health Care, Integrated/ |
| 89 | ((care or healthcare or health care or service?) adj3 (deliver* or integrat* or system or systems)).ti,kw,kf. |
| 90 | ((care or healthcare or health care or service?) adj3 (deliver* or integrat* or system or systems)).ab. /freq=2 |
| 91 | exp Health Services Accessibility/ |
| 92 | Health Resources/ |
| 93 | ((care or healthcare or health care) adj3 (access* or availab* or resourc* or right or rights)).tw,kw,kf. |
| 94 | Models, Organizational/ |
| 95 | (organi#ational adj2 model?).tw,kw,kf. |
| 96 | (model? adj3 (care or healthcare or health care or service?)).tw,kw,kf. |
| 97 | or/87-96 [**SERVICE DELIVERY/CARE MODELS**] |
| 98 | 8 and 97 [**FERTILITY/INFERTILITY - SERVICE DELIVERY/CARE MODELS**] |
| 99 | 44 or 50 or 72 or 86 or 98 [**FERTILITY/INFERTILITY - ALL ASPECTS**] |
| 100 | exp Animals/ not Humans/ |
| 101 | 99 not 100 [**ANIMAL-ONLY POPULATION REMOVED**] |
| 102 | (animal? or mice or mus or musculus or mouse or murine or woodmouse or woodmice or apodemus or rats or rat or rattus or murinae or muridae or cottonrat? or porcellus or hamster? or cricetinae or rodent$2 or porcine or pigs or pig or swine? or piglet? or boar or boars or sus or scrofa or ferret? or polecat? or mustela or putorius or norvegicus or guineapig? or guinea pig? or cavia or callithrix or marmoset? or cebuella or hapale or octodon or chinchilla? or gerbillinae or gerbil? or mesocricetus or cricetulus or cricetus or jird or jirds or merione? or unguiculatus or lagomorph or lagomorpha or rabbit? or oryctolagus or cuniculus or laprine or hares or hare or diptera or flies or fly or dipteral or drosphila or drosophilidae or insect? or feline or cats or cat or catus or felis or nematod$2 or sipunculida or dogs or dog or canine? or canis or lamb or lambs or sheep? or mouflon? or ovis or goats or goat or capra or capras or caprine or rupicapra or leporidae or chamois or haplorhini or glareolus or primate? or simian? or monkey? or macaque? or macaca? or mulatta? or cynomolgus or fascicularis or ateles or baboon? or papio or saimiri? or chlorocebus or vervet? or pygerythrus or anthropoidea or anthropoid? or saguinus or tamarin? or saguinus or leontopithecus or douroucoulis or aotus or hominidae or hominoidea or ape or apes or pan or hylobatidae or paniscus or bonobo? or troglodyte? or gibbon? or siamang? or nomascus or symphalangus or chimpanzee? or prosimian? or bushbab* or "bush baby" or bush babies or galago? or pongidae or gorilla? or pongo or pygmaeus or orangutan? or lemur or lemurs or lemuridae or loris or halichoerus or horse or horses or equus or equine or equidae or bovine or cow or cows or calf or bos taurus or bos indicus or bull or bulls or cattle or chicken? or gallus or quail? or aves or bird or birds or eagle or eagles or poultry or poultries or fowl or fowls or triturus or reptile? or reptilia? or bearded dragon? or pogona or vitticeps or anguis fragilis or snakes or snake or lizard? or alligator? or crocodile? or turtle or turtles or amphibia$2 or anura or frog or frogs or bombina or bufo or rana or salientia or toad or toads or epidalea or calamita or salamander? or newt or newst or killifish$2 or eel or eels or fish or fishes or pisces or catfish$2 or siluriformes or silurus or arius or heteropneustes or sheatfish$2 or clarias or gariepinus or perch or perches or percidae or perca or cichlidae or trout or trouts or char or chars or salvelinus or minnow? or cyprinidae or morhua or carps or carp or zebrafish$2 or danio or rerio or seabass$2 or sea bass$2 or dicentrarchus or labrax or morone or goldfish$2 or carassius or oncorhynchus or guppy or guppies or millionfish$2 or chub or chubs or tinca or barbels or barbus or pimephales or promelas or poecilia or "poecilia reticulata" or auratus or mullet? or sea horse? or seahorse? or mugil or curema or atlantic cod or atlantic cods or gadus or shark? or catshark? or cyprinus or carpio or anguilla or zander or sander or salmo or salmonid? or whitefish$2 or salmon? or sole or solea or lamprey? or petromyzon or lucioperca or pumpkinseed? or lepomis or gibbosus or herring? or clupea or harengus or sunfish$2 or tilapia? or oreochromis or sarotherodon or stizostedion or turbot? or psetta or flatfish$2 or plaice or pleuronectes or platessa or sciuridae or squirrel? or sciurus or chipmunk? or marmot? or suslik? or spermophilus or cynomys or sigmodon or vole or voles or microtus or myodes or lemming? or muskrat? or lemmus or otter? or lutra or meles or foumart or foulmart or marten? or martes or weasel? or badger? or ermine? or mink or minks or sable or sables or gulo or gulos or wolverine? or mustela or llama? or lama or lamas or glama or glamas or alpaca? or camelid or camelids or guanaco or guanaos or chiroptera? or coturnix or bobwhite? or colinus or virginianus or zebra finch$2 or taeniopygia or guttata or canary or canaries or serinus or canaria or grasskeet? or psittacine? or shelduck? or tadorna or goose or geese or branta or leucopsis or woodlark? or lullula? or flycatcher? or ficedula or hypoleuca or dove or doves or geopelia or cuneata or duck or ducks or graylag? or graylag? or anser or harrier? or circus pygargus or red knot? or great knot? or calidris or canutus or godwit? or limosa or lapponica or meleagris or gallopavo or jackdaw? or corvus or monedula or ruff or ruffs or philomachus or pugnax$2 or lapwing? or peewit? or plover? or vanellus or swan or swans or cygnet? or cygnus or columbianus or bewickii or gull or gulls or chroicocephalus or ridibundus or albifrons or great tit? or parus or aythya or fuligula or streptopelia or risoria or spoonbill? or platalea or leucorodia or blackbird? or turdus or merula? or blue tit? or cyanistes or pigeon? or columba or pintail? or anas or starling? or sturnus or owl or owls or athene noctua or pochard or ferina or cockatiel or nymphicus or hollandicus or skylark? or alauda or tern or terns or sterna or teal or crecca or oystercatcher? or haematopus or ostralegus or sorex or araneus or crocidura or russula or moles or mole or talpa or bat or bats or eptesicus or serotinus or myotis or dasycneme or daubentonii or pipistrelle or pipistrellus or fox or foxes or vulpes or common seal? or phoca or vitulina or grey seal? or iguana? or xenopus or laevis or parakeet? or parrot? or donkey? or mule or mules or zebra? or shrew or shrews or bison or bisons or buffalo$2 or deer or deers or cervus or elaphus or bear or bears or panda or pandas or hog or hogs or boar or boars or fitchew? or fitch$2 or beaver? or castor fiber? or castor canadensis or jerboa? or jaculus or capybara? or marsupial*).ti,kw,kf. |
| 103 | 101 not 102 [**ANIMAL RECORDS - TITLE LEVEL - REMOVED**] |
| **104** | **limit 103 to yr="1990-current" [1990 DATE OPTION]** |
